# Effective removal of host cell-derived nucleic acids bound to hepatitis B core antigen virus-like particles by heparin chromatography

**DOI:** 10.3389/fbioe.2024.1475918

**Published:** 2024-10-03

**Authors:** Angela Valentic, Jürgen Hubbuch

**Affiliations:** Institute of Process Engineering in Life Sciences – Section IV: Biomolecular Separation Engineering, Karlsruhe Institute of Technology (KIT), Karlsruhe, Germany

**Keywords:** virus-like particles, heparin chromatography, HBcAg, host cell-derived nucleic acids, process development

## Abstract

Virus-like particles (VLPs) show considerable potential for a wide array of therapeutic applications, spanning from vaccines targeting infectious diseases to applications in cancer immunotherapy and drug delivery. In the context of hepatitis B core antigen (HBcAg) VLPs, a promising candidate for gene delivery approaches, the naturally occurring nucleic acid (NA) binding region is commonly utilized for effective binding of various types of therapeutic nucleic acids (NA_ther_). During formation of the HBcAg VLPs, host cell-derived nucleic acids (NA_hc_) might be associated to the NA binding region, and are thus encapsulated into the VLPs. Following a VLP harvest, the NA_hc_ need to be removed effectively before loading the VLP with NA_ther_. Various techniques reported in literature for this NA_hc_ removal, including enzymatic treatments, alkaline treatment, and lithium chloride precipitation, lack quantitative evidence of sufficient NA_hc_ removal accompanied by a subsequent high VLP protein recovery. In this study, we present a novel heparin chromatography-based process for effective NA_hc_ removal from HBcAg VLPs. Six HBcAg VLP constructs with varying lengths of the NA binding region and diverse NA_hc_ loadings were subjected to evaluation. Process performance was thoroughly examined through NA_hc_ removal and VLP protein recovery analyses. Hereby, reversed phase chromatography combined with UV/Vis spectroscopy, as well as silica spin column-based chromatography coupled with dye-based fluorescence assay were employed. Additionally, alternative process variants, comprising sulfate chromatography and additional nuclease treatments, were investigated. Comparative analyses were conducted with LiCl precipitation and alkaline treatment procedures to ascertain the efficacy of the newly developed chromatography-based methods. Results revealed the superior performance of the heparin chromatography procedure in achieving high NA_hc_ removal and concurrent VLP protein recovery. Furthermore, nuanced relationships between NA binding region length and NA_hc_ removal efficiency were elucidated. Hereby, the construct Cp157 surpassed the other constructs in the heparin process by demonstrating high NA_hc_ removal and VLP protein recovery. Among the other process variants minimal performance variations were observed for the selected constructs Cp157 and Cp183. However, the heparin chromatography-based process consistently outperformed other methods, underscoring its superiority in NA_hc_ removal and VLP protein recovery.

## 1 Introduction

Virus-like particles (VLPs) are extensively exploited for a vast variety of therapeutic applications (Boisgérault et al., 2002; Mohsen et al., 2017; Rohovie et al., 2017; Nooraei et al., 2021). Vaccines using VLP technology against certain types of human papillomavirus (HPV), preventing cervical cancer, other HPV-related diseases, and hepatitis B virus infections are already on the market (Mohsen et al., 2017; Zhao et al., 2020; Illah and Olaitan, 2023). In the context of VLP-based vaccine development, there are ongoing efforts for cancer immunotherapy, engineering VLPs to display tumor-associated antigens, thus triggering an immune response against cancer cells (Klamp et al., 2011; Li et al., 2021). In this context, a hepatitis B core antigen (HBcAg) based vaccine recently demonstrated potency to induce humoral and cell-mediated immune responses against SARS-CoV-2 infection (Hassebroek et al., 2023). By incorporating therapeutic payloads into VLPs, they can potentially be used as drug delivery vehicles, thereby protecting and delivering therapeutic components to target cells (Cooper and Shaul, 2005; Porterfield et al., 2010; Choi et al., 2013; Jinming et al., 2013; Rohovie et al., 2017; Fang et al., 2018; Petrovskis et al., 2021). There is for example research on VLP engineering to display specific targeting ligands on the surface allowing for targeted drug delivery to specific cells or tissues, or combining VLPs with other materials, such as lipids or synthetic polymers, to enhance stability, payload capacity, and drug release characteristics of these hybrid VLPs (Hill et al., 2017; Mohsen et al., 2017; Rohovie et al., 2017).

**FIGURE 1:**
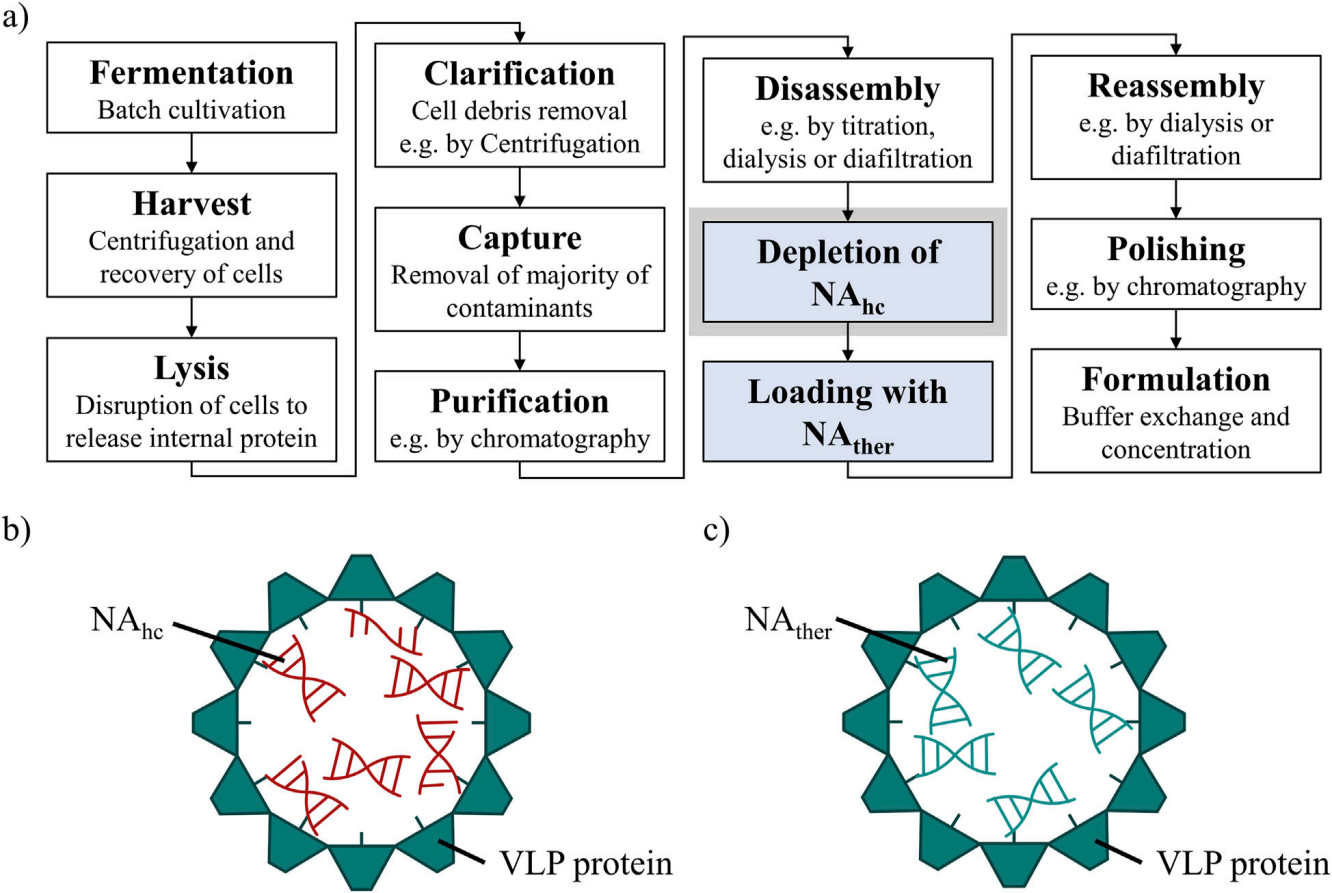
**(A)** Common HBcAg VLP purification process (Hillebrandt et al., 2021) with depletion of NA_hc_ and loading with NA_ther_, process steps required for the application as a nucleic acid delivery vector, highlighted in blue. The depletion of NA_hc_ investigated in this study is highlighted in grey. **(B)** HBcAg VLP with NA binding region and NA_hc_ encapsulated within the VLP protein capsid. **(C)** HBcAg VLP with NA binding region and NA_ther_ encapsulated within the VLP protein capsid after depletion of NA_hc_ and loading with NA_ther_. HBcAg: hepatitis B core antigen, NA: nucleic acids, NA_hc_: host cell-derived nucleic acids, NA_ther_: therapeutic nucleic acids, VLP: virus-like particle.

However, the effective downstream processing of VLPs continues to be a major challenge. The purification process after intracellular formation of VLPs in an expression system, such as *E. coli*, yeast or plant cells (Effio and Hubbuch, 2015), typically involves cell lysis, clarification, precipitation or ultracentrifugation, disassembly and reassembly, followed by polishing and formulation (McCarthy et al., 1998; Zhao et al., 2012; Hillebrandt et al., 2020; Zhang et al., 2021). Recent advancements to enhance efficiency and scalability for VLP purification processes, using ultrafiltration-based unit operations, might be applied (Negrete et al., 2014; Carvalho et al., 2019; Hillebrandt et al., 2020; 2021; Hillebrandt and Hubbuch, 2023). However, for gene delivery, VLP purification processes require additional steps (Figure 1A) such as the loading of VLPs with therapeutic nucleic acids (NA_ther_) (Rohovie et al., 2017; Valentic et al., 2022). In the case of the HBcAg VLP, a promising candidate for gene delivery applications, the naturally occurring nucleic acid (NA) binding region is commonly utilized for effective binding of various types of NA_ther_. The wild-type HBcAg protein with the full-length NA binding region (Nassal, 1992; Zhang et al., 2021), named Cp183 in the course of this manuscript, and variants with several amino acid modifications (Porterfield et al., 2010; Strods et al., 2015), as well as HBcAg VLP constructs with different lengths of this naturally occurring NA binding region (Newman et al., 2009; Liu et al., 2010; Sominskaya et al., 2013; Petrovskis et al., 2021; Valentic et al., 2022) are employed. To load VLPs with NA_ther_, the disassembled and purified VLP subunits, so called dimers, are mixed with NA_ther_ and then reassembled into loaded VLPs (Figure 1C). However, during initial formation of the HBcAg VLPs in the *E. coli* cells, host cell-derived nucleic acids (NA_hc_) associate also to the NA binding region and are encapsulated into the VLPs (Birnbaum and Nassal, 1990; Newman et al., 2009; Porterfield et al., 2010) (Figure 1B). These NA_hc_ influence the subsequent downstream processing (Valentic et al., 2022) and obstruct the desired binding of NA_ther_ during loading. An effective removal of the undesired NA_hc_ bound to the HBcAg VLP NA binding region is required both to prevent potential side effects due to the presence of NA_hc_ and to subsequently load the VLPs with NA_ther_.

There are several techniques reported in literature that attempt to remove NA_hc_ using different approaches. Enzymatic treatments (Newman et al., 2009), sometimes coupled with His-tag affinity chromatography (Zhang et al., 2021), are applied to degrade and separate undesired NA_hc_. Though, nuclease treatment is expensive and a thorough removal of the nuclease in the subsequent purification steps is essential prior to loading with NA_ther_. In addition, only proteins with His-tag can be purified using His-tag affinity chromatography. Besides, extensive alkaline treatment is used in combination with ammonium sulfate precipitation to separate NA_hc_ (Strods et al., 2015). However, alkaline treatment may cause pH stress to the proteins, while being time and buffer extensive. Furthermore, Lithium chloride precipitation is used to precipitate encapsulated RNA during disassembly of the VLPs (Porterfield et al., 2010). However, the effects on bound DNA are uncertain and the use of guanidine HCl and LiCl as part of the disassembly and precipitation liquid phase is laborious and expensive.

In these studies, the removal of NA_hc_ is evaluated qualitatively by transmission electron microscopy, native agarose gel electrophoresis (NAGE) and A260/A280 ratios gained by UV/Vis spectroscopy. Quantitative analysis of NA_hc_ removal with concurrent high VLP protein recovery, which is essential for effective downstream purification processing, is missing. Only recently, novel quantification methods for the absolute quantification of HBcAg VLP proteins and bound nucleic acids were presented by our group (Valentic et al., 2024). This allows a thorough examination of NA_hc_ removal and VLP protein recovery for to evaluate the performance of different NA_hc_ removal processes.

Next to the above-mentioned methods used for NA_hc_ removal, heparin affinity chromatography appears to be a promising approach for efficient NA_hc_ removal. It has already been used for the purification of viruses (Nasimuzzaman et al., 2016; Spannaus et al., 2017) and VLPs (Minkner et al., 2018; Reiter et al., 2019; Zollner et al., 2024). During the purification of HIV-1 gag virus-like particles, both extracellular vesicles and chromatin were separated (Pereira Aguilar et al., 2020). In the case of HBcAg VLPs, studies described the interaction with cell-surface-expressed heparan sulphate by the protamine-like NA binding region (Cooper et al., 2005; Vanladschoot et al., 2005). Moreover, the binding of the NA binding region of HBcAg VLPs to heparin chromatographic media was demonstrated by Broos et al., 2007. This is why heparin chromatography exhibits great potential for effective removal of NA_hc_ bound to HBcAg VLPs.

In this work, we present a novel heparin chromatography-based process for an effective removal of NA_hc_ bound to HBcAg VLPs. The heparin chromatography step was examined with six different HBcAg VLP constructs with varying lengths of the NA binding region possessing different NA_hc_ loadings. Process performance was evaluated by a quantitative determination of NA_hc_ removal and VLP protein recovery with reversed phase (RP) chromatography coupled with UV/Vis analysis, and silica spin column (SC) based chromatography followed by dye-based fluorescence assay as described earlier (Valentic et al., 2024). Further, different process variants including sulfate chromatography instead of heparin chromatography and nuclease treatments prior to these chromatographic techniques were investigated. In addition, LiCl precipitation and alkaline treatment procedures were performed according to literature. Process performances were determined enabling a thorough comparison of the various removal techniques. For the comparison of different process variants and procedures, Cp157, a construct with an intermediate length of the NA binding region, and Cp183, one with the full-length NA binding region, were examined. For all processes, efficacy was evaluated by a quantitative determination of NA_hc_ removal and VLP protein recovery at different process stages. In addition, A260/A280 ratios were determined by UV/Vis spectroscopy to assess the VLP protein purity. SDS-PAGE and NAGE analyses were carried out to examine the VLP protein and nucleic acid content at various stages of the processes to complement the comprehensive comparative study.

## 2 Materials and methods

### 2.1 Buffer and VLPs

All chemicals were purchased from Merck (Darmstadt, Germany), if not stated otherwise. Solutions and buffers were prepared with ultrapure water (PURELAB Ultra, ELGA LabWater) and aqueous buffers were filtered through a 0.2 µm pore-size cellulose acetate filter (Pall Corporation, Port Washington, NY, United States). Buffers were pH-adjusted with 4 M NaOH or 32% HCl. Trifluoroacetic acid (TFA) was purchased from Thermo Fisher Scientific (Waltham, MA, United States) and HPLC grade acetonitrile (ACN) from Avantor (Radnor, PA, United States). HBcAg VLPs with different lengths of nucleic acid binding regions and different amounts of bound host cell-derived nucleic acids (NA_hc_) were produced and purified as described earlier (Valentic et al., 2022). Constructs Cp149, Cp154, Cp157, Cp164, Cp167, and Cp183 (Valentic et al., 2022) after CaptoCore 400 purification and present in purification buffer consisting of 50 mM Tris and 150 mM NaCl at pH 7.2 were used for all experiments.

### 2.2 Removal of host cell-derived nucleic acids bound to HBcAg VLPs

**FIGURE 2:**
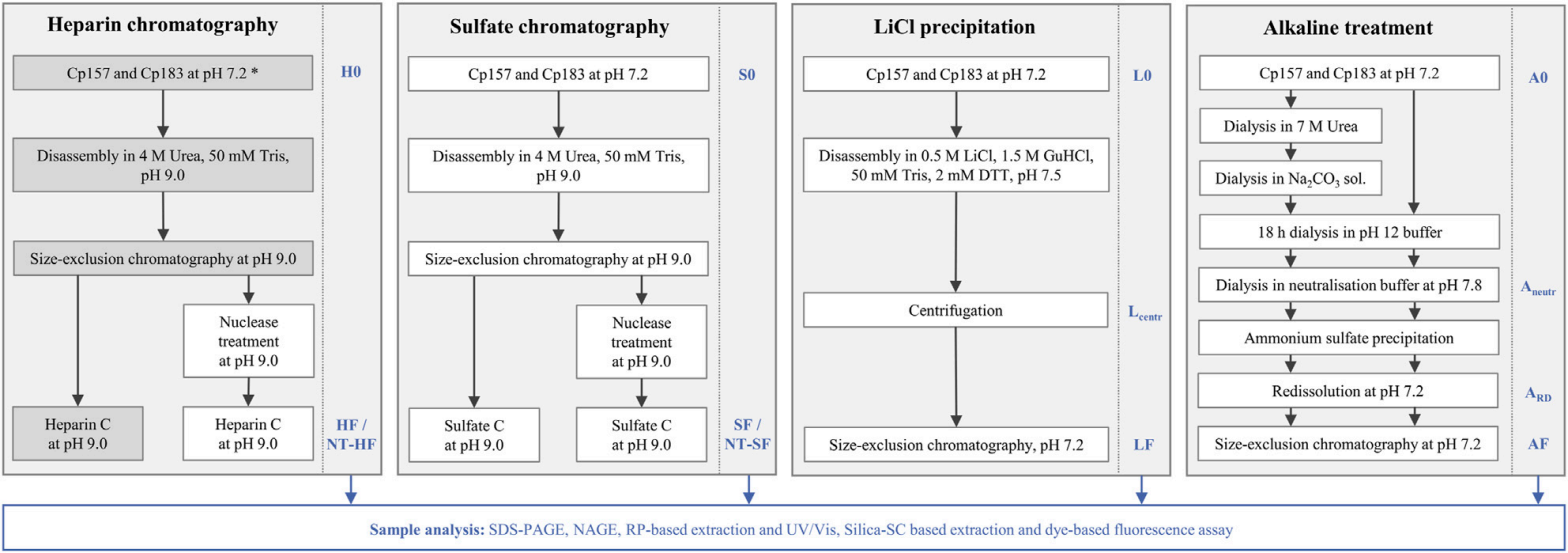
Overview of processes investigated in this work to remove host cell-derived nucleic acids bound to HBcAg VLPs. Heparin and sulfate chromatography were performed with and without prior nuclease treatment. LiCl precipitation and alkaline treatment were adapted from reported techniques in literature (Porterfield et al., 2010; Petrovskis et al., 2021). The alkaline treatment procedure was performed with and without the reported preliminary dialysis in 7 M Urea and NaCO_3_ solutions. All outlined removal techniques were performed with Cp157 and Cp183 HBcAg VLP constructs (Valentic et al., 2022), while the grey shaded process path was additionally performed for Cp149, Cp154, Cp164, and Cp167 (*). Initial, intermediate and final samples, which are labelled at the process steps, respectively, were analysed by SDS-PAGE, NAGE, RP chromatography coupled with UV/Vis analysis, and silica-SC based chromatography followed by dye-based fluorescence assay (Valentic et al., 2024). HBcAg: hepatitis B core antigen, NAGE: native agarose gel electrophoresis, NT: nuclease treatment, RP: reversed phase, VLP: virus-like particle.

All removal techniques were performed in duplicates with Cp157 and Cp183 HBcAg VLP constructs, while the heparin chromatography without prior nuclease treatment was additionally performed for Cp149, Cp154, Cp164, and Cp167. An overview about the investigated removal techniques and VLP constructs can be found in Figure 2.

#### 2.2.1 Heparin and sulfate chromatography

HBcAg VLP capsids were disassembled overnight at 4°C by dilution with 50 mM Tris, 10 M Urea stock solution to a final concentration of 4 M Urea, 50 mM Tris and titrated to pH 9.0. After filtration by a 0.2 µm cellulose acetate filter, dimers were purified by size-exclusion chromatography (SEC) using a 150 mL Toyopearl HW-65S column equilibrated in disassembly buffer (50 mM Tris, 4 M Urea, pH 9.0). To remove bound NA_hc_, the dimer fractions were purified on a 1 mL HiTrap™ Heparin HP (Cytiva, Marlborough, MA, United States) column or a 5 mL Toyopearl Sulfate-650F (Tosoh Bioscience, Tokio, Japan) column equilibrated in disassembly buffer, with or without prior nuclease treatment with DENARASE^®^ (c-LEcta GmbH, Leipzig, Germany). Elution was performed with a high-salt buffer (50 mM Tris, 4 M Urea, 1 M NaCl, pH 9.0). Flow-through and elution fractions were analyzed by SDS-PAGE, RP chromatography coupled with UV/Vis analysis, and silica spin column (SC) based chromatography followed dye-based fluorescence assay.

#### 2.2.2 LiCl precipitation

The LiCl precipitation was performed in accordance with the literature, with few adaptions (Porterfield et al., 2010). In brief, the HBcAg VLP capsids were disassembled with 0.5 M LiCl, 1.5 M guanidine HCl 0.05 M Tris, 2 mM DTT, pH 7.5 overnight, followed by centrifugation for 20 min at 12,000 rpm at 4 °C and filtration of the supernatant with 0.2 µm cellulose acetate filter. SEC of the filtered supernatant was performed with a 25 mL Superose 6 Increase 10/300 GL (Cytiva, Marlborough, MA, United States) column equilibrated in redissolution buffer. Besides initial Cp157 and Cp183 material and final SEC fractions, intermediate process samples after centrifugation were analyzed. Samples were analyzed by SDS-PAGE, NAGE, RP chromatography coupled with UV/Vis analysis, and silica-SC based chromatography followed by dye-based fluorescence assay.

#### 2.2.3 Alkaline treatment

The alkaline treatment was performed in accordance with the literature, with a number of adaptions (Strods et al., 2015). In brief, the HBcAg VLP capsids were dialyzed in 7 M Urea for 16 h at 4 °C, followed by dialysis in 0.1 M Na_2_CO_3_, 2 mM DTT solution. For the alkaline treatment, the VLPs were dialyzed in alkaline buffer (0.1 M sodium phosphate, 0.65 M NaCl, pH 12) for 18 h at 4°C. After dialysis in neutralization buffer (0.1 M sodium phosphate, 0.65 M NaCl, pH 7.8) the VLPs were precipitated with 1 M AMS, centrifugated for 30 min, at 12,000 rpm and 4°C, and re-dissolved overnight with purification buffer at 4°C. After centrifugation for 30 min at 6,000 rpm and filtration of supernatant with 0.2 µm cellulose acetate filter, SEC was performed with a 25 mL Superose 6 Increase 10/300 GL (Cytiva, Marlborough, MA, United States) column equilibrated in redissolution buffer. In parallel, alkaline treatment, precipitation, and SEC were performed without prior dialysis into 7 M Urea and Na_2_CO_3_ solutions. Besides initial Cp157 and Cp183 material and final SEC fractions, intermediate process samples after neutralization and after redissolution were analyzed. Samples were analyzed by SDS-PAGE, NAGE, RP chromatography coupled with UV/Vis analysis, and silica-SC based chromatography followed by dye-based fluorescence assay.

### 2.3 Analytics

#### 2.3.1 SDS-PAGE

For SDS-PAGE, NuPage 4%–12% BisTris protein gels, MES running buffer, and LDS sample buffer were used. Gels were run on a PowerEase Touch 350W Power Supply (all Invitrogen, Waltham, MA, United States) at reduced mode with 50 mM DTT in the sample solution. Coomassie blue solution was used for protein staining.

#### 2.3.2 NAGE

For NAGE, 0.7% agarose (Carl Roth, Karlsruhe, Germany) in TAE buffer (40 mM Tris, 20 mM acetic acid, 1 mM EDTA) with 1 μg/mL midori green (Nippon Genetics GmbH, Düren, Germany) was used. Gels were run on a PowerPac Basic (Bio-Rad, Hercules, CA, United States). Coomassie blue solution was used for subsequent protein staining, if necessary.

#### 2.3.3 Protein and nucleic acid quantification

RP chromatography coupled with UV/Vis analysis was used to separate and quantify the bound NA_hc_ and VLP proteins in accordance to an earlier publication of our group (Valentic et al., 2024). Hereby, an analytical reversed-phase chromatography was performed with a TSKgel Protein C4-300 column (3 μm, 4.6 × 150 mm) from Tosoh Bioscience (Tokyo, Japan) on a Vanquish UHPLC system, controlled by Chromeleon version 7.2 (both Thermo Fisher Scientific, Waltham, MA, Unites States). In parallel, a silica-SC based chromatography followed dye-based fluorescence assay was used to quantify NA_hc_, according to a previous publication of our group (Valentic et al., 2024). Hereby, materials included in the EasyPure Viral DNA/RNA Kit (TransGen Biotech, Beijing, China) were used for nucleic acid extraction and RiboGreen assay (Thermo Fisher Scientific, Waltham, MA, Unites States) was performed for NA_hc_ quantification according to the manufacturer’s manual with minor adaptations.

To calculate the nucleic acid removal and VLP protein recovery, the respective nucleic acid or VLP protein mass of the chromatography fraction or intermediate sample was divided by the initial nucleic acid or VLP protein mass for all performed quantification techniques. Chromatography fractions were the elution fractions at heparin and sulfate chromatography and the final SEC fractions for the LiCl precipitation and alkaline treatment. NA_hc_ removal results deviated for the two utilized analytical methods. Further, nucleic acid quantification by RP-UV/Vis of intermediate LiCl precipitation samples after centrifugation was not possible due to the presence of guanidine HCl in the samples, overlapping with the nucleic acid peaks in the flow-through of the RP-HPLC method. Therefore, it was decided to show the NA_hc_ removals obtained by the two available analytical methods independently. A260/A280 ratio of chromatography fractions were determined by analysis of chromatography peak areas at 260 nm and 280 nm. For initial material and intermediate process samples, the A260/A280 ratio was determined by microvolume UV/Vis absorbance measurements using a NanoDrop™ 2000c UV/Vis spectrophotometer (Thermo Fisher Scientific, Waltham, MA, Unites States).

## 3 Results

Effective processing for the removal of host cell-derived nucleic acids (NA_hc_) bound to hepatitis B core antigen (HBcAg) virus-like particles (VLPs) is defined by low final NA_hc_ concentrations accompanied with high VLP protein recoveries. NA_hc_ removals and VLP protein recoveries were quantitatively assessed by reversed phase (RP) chromatography coupled with UV/Vis analysis, and silica spin column (SC) based chromatography followed by dye-based fluorescence assay (Valentic et al., 2024). Due to discrepancies in the NA_hc_ removal results between the two analytical methods, and the interference caused by guanidine HCl in some LiCl precipitation samples during nucleic acid quantification by RP-UV/Vis, it was decided to report the NA_hc_ removal data from each method separately throughout the study. VLP protein purities were additionally evaluated by determination of A260/A280 ratios. To qualitatively track the VLP proteins and nucleic acids in the respective samples, SDS-PAGE and native agarose gel electrophoresis (NAGE) analyses were performed for all process samples. An overview of processes to remove NA_hc_, all the initial, intermediate and final process samples analyzed and their labeling is shown in Figure 2.

### 3.1 Heparin chromatography for different HBcAg VLP constructs

**FIGURE 3:**
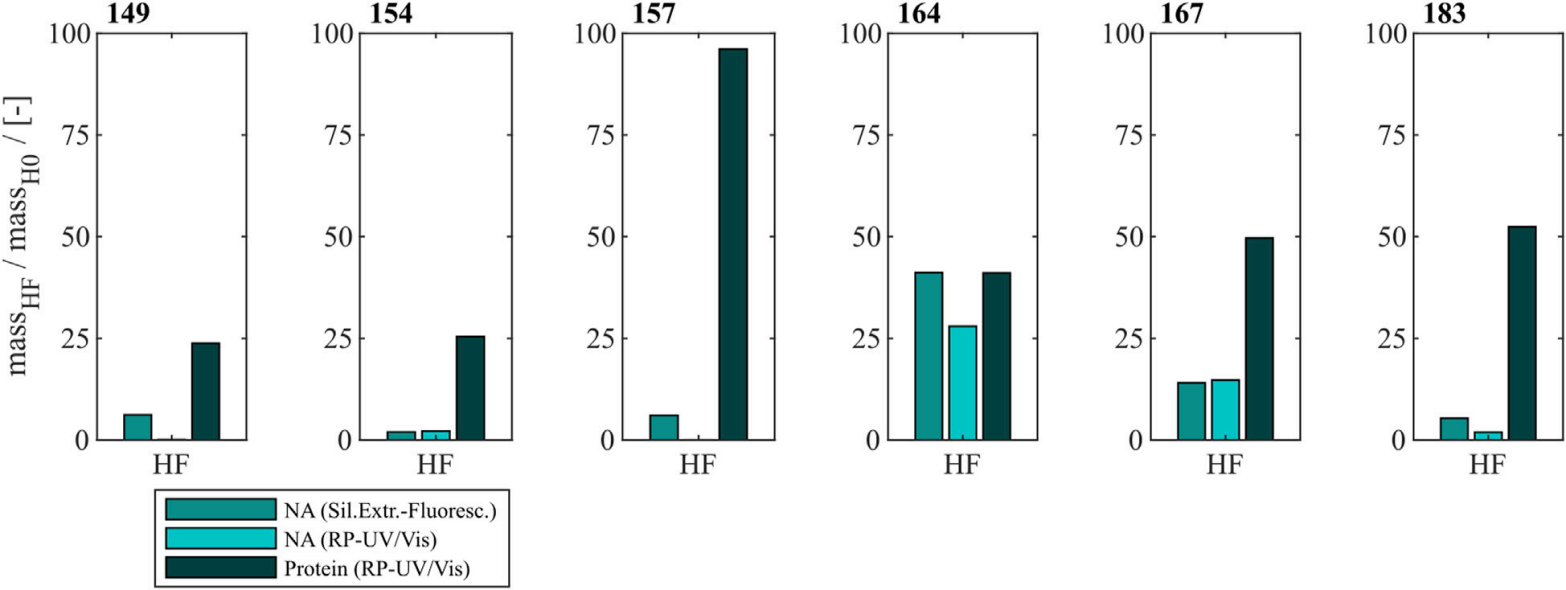
Protein and nucleic acid recoveries for six HBcAg VLP constructs after removal of host cell-derived nucleic acids by disassembly and heparin chromatography (highlighted process path in Figure 1). Nucleic acid recoveries were analysed by silica-SC based chromatography followed by dye-based fluorescence assay and RP chromatography coupled with UV/Vis analysis. VLP protein recoveries were determined by RP chromatography coupled with UV/Vis analysis. HBcAg: hepatitis B core antigen, NAGE: native agarose gel electrophoresis, RP: reversed phase, SC: spin column, VLP: virus-like particle.

To effectively remove NA_hc_ bound to HBcAg VLPs a heparin chromatography-based purification step was developed. Six different constructs with different lengths of a nucleic acid (NA) binding region and varying loads of NA_hc_ were investigated. The retention of VLP proteins during the heparin chromatography without previous nuclease treatment was examined by SDS-PAGE. The respective gel scans are depicted in Supplementary Material S1. Cp149 and Cp154 were mainly present in the flow-through fractions of the heparin chromatography. Cp157 and Cp183 showed clear binding and were thus detected in the elution fractions. SDS-PAGE results for Cp165 and Cp167 were inconclusive due to concentration restrictions or method errors. Further, the purity and recovery of the VLP proteins after the NA_hc_ removal step (grey shaded process path in Figure 2) were determined for all constructs and are depicted in Figure 3.

#### 3.1.1 Nucleic acid removal

For Cp149, Cp154, Cp157 and Cp183 the average nucleic acid removal was as high as 97%. For Cp164 and Cp167 the nucleic acid removal was less successful with NA_hc_ removal of around 86% for Cp167 and depending on the NA_hc_ quantification method either 58% (silica-SC based chromatography followed by dye-based fluorescence assay) or 72% (RP-UV/Vis) for Cp164.

#### 3.1.2 VLP protein recovery

However, the respective VLP protein recovery has to be considered, as high VLP protein recoveries are required for effective processing The VLP protein recovery for the heparin chromatography purification step mostly increases with the length of the NA binding region. VLP protein recoveries ranged from 24% for Cp149% to 52% for Cp183. Nevertheless, Cp157 showed the highest VLP protein recovery with 96%.

In total, Cp157 demonstrated a high nucleic acid removal accompanied with high protein recovery, as desired for an effective removal of NA_hc_ bound to HBcAg VLPs. Complementarily, the A260/A280 ratios were assessed as an indicator for VLP protein purity and can be found in Table 1. The A260/A280 ratios for the initial samples differed greatly between 0.63 for Cp149 and 2.08 for Cp164. For all six VLP constructs, a decreased A260/A280 ratio was determined after heparin chromatography without nuclease treatment compared to the ratio of the initial sample. Cp157 resulted in the lowest A260/A280 ratio of 0.55. In summary, heparin chromatography demonstrated divergent NA_hc_ removal performances depending on the VLP construct, with Cp157 resulting in high NA_hc_ removal and high VLP protein recovery.

**TABLE 1:** A260/A280 ratio of investigated HBcAg VLP constructs after removal of host cell-derived nucleic acids bound to HBcAg VLPs by heparin and sulfate chromatography without (w/o) and with (w) prior nuclease treatment and initial material. Ratios were determined by analysis of selected chromatography peak areas at 260 nm and 280 nm, or by microvolume UV/Vis absorbance measurements for initial samples.

	Nuclease treatment	A260/A280 [-]					
		Cp149	Cp154	Cp157	Cp164	Cp167	Cp183
Initial Sample		0.63	1.94	1.24	2.08	1.63	1.74
Heparin chromatography	w/o	0.62	0.74	0.57	0.67	0.66	0.71
	w	—	—	0.55	—	—	0.69
Sulfate chromatography	w/o	—	—	0.62	—	—	0.63
	w	—	—	0.57	—	—	0.59

### 3.2 Heparin and sulfate chromatography with and without prior nuclease treatment

**FIGURE 4:**
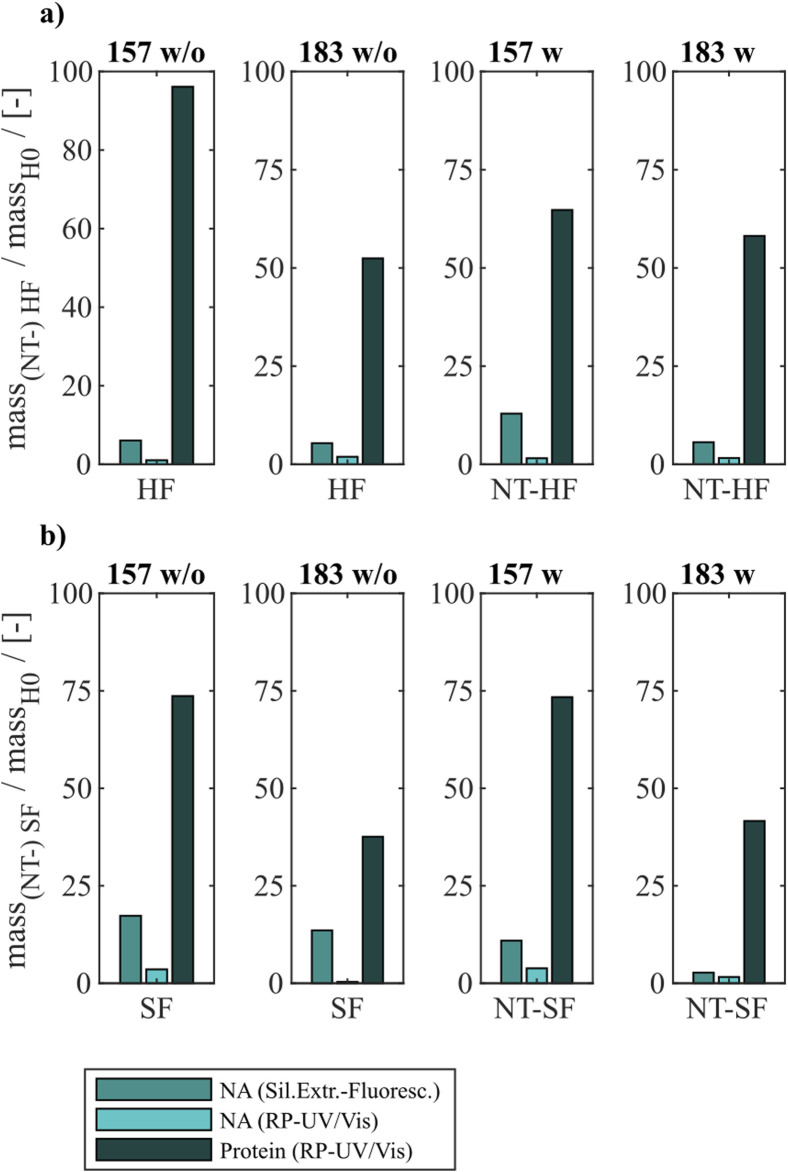
VLP protein and nucleic acid recoveries for Cp157 and Cp183 after removal of host cell-derived nucleic acids by disassembly, with (w) or without (w/o) prior nuclease treatment and **(A)** heparin chromatography or **(B)** sulfate chromatography. Nucleic acid recoveries were analysed by silica-SC based chromatography followed by dye-based fluorescence assay and RP chromatography coupled with UV/Vis analysis. VLP protein recoveries were determined by RP chromatography coupled with UV/Vis analysis. RP: reversed phase, SC: spin column.

Different chromatography-based process variants were investigated regarding their NA_hc_ removal performance for Cp157 and Cp183. In addition to heparin chromatography, sulfate chromatography was investigated. Moreover, both chromatographic techniques were performed with and without nuclease treatment and the results for NA_hc_ removal and VLP protein recovery are displayed in Figure 4. The presence of VLP proteins in the elution fractions of both chromatography-based techniques were verified by SDS-PAGE. The respective gel scans are depicted in Supplementary Material S2.

For all investigated process variants averaged nucleic acid removals of at least 90% were achieved. This was confirmed by decreased A260/A280 ratios after NA_hc_ removal compared to the ratio of the initial samples (Table 1). However, the VLP protein recoveries ranged from 96% (Cp157, heparin chromatography, without nuclease treatment) to 38% (Cp183, sulfate chromatography, without nuclease treatment).

#### 3.2.1 Constructs

Cp157 showed higher VLP protein recoveries than Cp183 with each chromatography-based process variant, while NA_hc_ removal with Cp157 showed to be comparable to Cp183. In addition, lower A260/A280 ratios were achieved for Cp157 than for Cp183.

#### 3.2.2 Nuclease treatment

In general, nuclease treatment improved NA_hc_ removal performance for the process variants investigated by up to 4.8% (for Cp183 after subsequent sulfate chromatography). However, for Cp157 and subsequent heparin chromatography, the process with nuclease treatment achieved lower NA_hc_ removals than the process without nuclease treatment. VLP protein recoveries were not affected by the nuclease treatment, except for the heparin chromatography with Cp157. There, a lower VLP protein recovery was detected for the process with nuclease treatment. Purities evaluated by A260/A280 ratios were improved by nuclease treatment for all process variants investigated (see Table 1).

#### 3.2.3 Heparin and sulfate chromatography

Heparin chromatography mostly achieved higher NA_hc_ removals and VLP protein recoveries than sulfate chromatography. An exception of this was the process investigating Cp157 with nuclease treatment, where a lower VLP protein recovery was detected with heparin chromatography than with sulfate chromatography. Nevertheless, in terms of purity, heparin chromatography achieved lower A260/A280 ratios for Cp157, as for Cp183, throughout all process variants investigated.

In summary, the best removal performances with high NA_hc_ removal, high VLP protein recovery and high VLP protein purity were achieved for Cp157 by heparin chromatography with and without prior nuclease treatment.

### 3.3 LiCl precipitation

**FIGURE 5:**
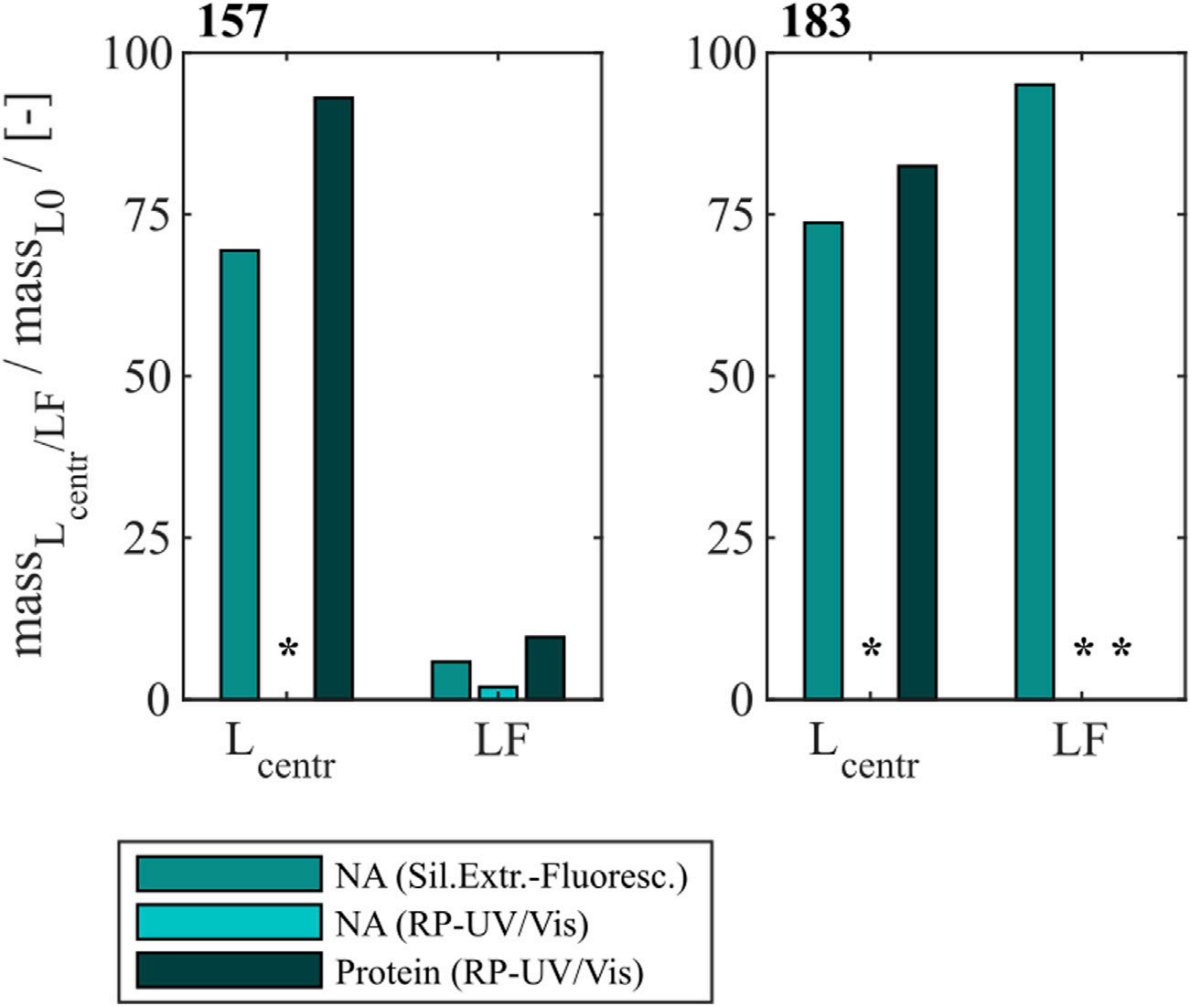
VLP protein and nucleic acid recoveries for Cp157 and Cp183 after removal of host cell-derived nucleic acids by disassembly and LiCl precipitation, centrifugation (intermediate sample) and size-exclusion chromatography. Nucleic acid recoveries were analysed by silica-SC based chromatography followed by dye-based fluorescence assay and RP chromatography coupled with UV/Vis analysis. VLP protein recoveries were determined by RP chromatography coupled with UV/Vis analysis. Nucleic acid quantification by RP-UV/Vis was not possible after centrifugation due to the presence of guanidine HCl in the samples, overlapping with the nucleic acid peaks in the flow-through of the RP-HPLC method, and nucleic acid and VLP protein concentrations of Cp183 in SEC fraction were too low for RP-UV/Vis analysis (*). RP: reversed phase, SC: spin column, SEC: size-exclusion chromatography.

To compare the developed heparin chromatographic procedure with the LiCl precipitation with respect to their NA_hc_ removal performance, LiCl precipitation was performed for Cp157 and Cp183. An overview of initial, intermediate and final process samples analyzed and their labeling is shown in Figure 2. Samples after disassembly, LiCl precipitation and centrifugation (L1), and samples after size-exclusion chromatography (SEC) (LF) were analyzed. Chromatograms of the final SEC and fraction segmentation for analysis can be found in Supplementary Material S3. The fraction pools with the highest expected VLP protein purity, estimated from the A260/A280 ratio of the peak areas in the chromatogram, were selected for analysis of NA_hc_ removal and VLP protein recovery. The levels of NA_hc_ removal and VLP protein recovery for the fraction pool #3 for Cp157 and fraction pool #1 for Cp183 are depicted in Figure 5.

For Cp157, a NA_hc_ removal of 31% with 93% VLP protein recovery was determined after centrifugation (L1). After SEC the dimer comprising fractions with the lowest A260/A280 values were analyzed. This revealed a high VLP protein loss of 90% with a nucleic acid removal of 96% (LF). Similar results were detected for Cp183, achieving 26% NA_hc_ removal with 82% VLP protein recovery after centrifugation (L1). Again, the SEC revealed high VLP protein loss, resulting in nucleic acid and VLP protein concentrations too low for RP-UV/Vis analysis (LF). On the contrary, the performed silica-SC based chromatography followed by dye-based fluorescence assay resulted in a lower NA_hc_ removal of 5% than formerly determined for the centrifugation sample (L1). This might be caused by assay imprecision due to the low concentrations. Guanidine HCl overlaps the nucleic acid peaks in the flow-through of the used RP-HPLC method. Therefore, nucleic acid quantification by RP-UV/Vis was not possible due to the presence of guanidine HCl in the samples after centrifugation.

The complementary analysis of HBcAg VLP protein purity by A260/A280 ratios listed in Table 2 underline these findings. It shows low VLP protein purities in the SEC fractions for both Cp157 and Cp183, with A260/A280 ratios of 0.95 and 1.74, respectively. SDS-PAGE and NAGE analyses were performed for all process samples to qualitatively track the VLP proteins and nucleic acids in the respective samples. Gel scans are depicted in Supplementary Material S3. Gel-derived results mainly support the findings for NA_hc_ removals and VLP protein recoveries described above. However, SDS-PAGE displayed a greatly attenuated Cp157 lane for samples after centrifugation (L1) compared to the initial sample. This indicates VLP protein loss during LiCl precipitation and subsequent centrifugation, unlike 93% VLP protein recovery for this sample described above. In NAGE analysis shifted VLP lanes and an additional strong free nucleic acid lane were visible, compared to the initial sample. Similar results were found for Cp183 in SDS-PAGE and NAGE, but less pronounced due to low concentrations. Overall, LiCl precipitation resulted in high VLP protein losses during processing and low final VLP protein purities. VLP protein recovery of Cp157 was 86% lower than with heparin chromatography.

**TABLE 2:** A260/A280 ratio of Cp157 and Cp183 samples after removal of host cell-derived nucleic acids bound to HBcAg VLPs by LiCl precipitation and intermediate samples and initial material. Ratios were determined by analysis of selected chromatography peak areas at 260 nm and 280 nm, or by microvolume UV/Vis absorbance measurements for initial and intermediate samples. Ratios of samples after centrifugation might be affected by the presence of guanidine HCl in the samples and are listed in brackets.

	A260/A280 [-]					
	Cp157			Cp183		
LiCl precipitation	initial	centr	SEC frac	initial	centr	SEC frac
	1.33	(1.24)	0.95	1.93	(1.76)	1.74

### 3.4 Alkaline treatment

**FIGURE 6:**
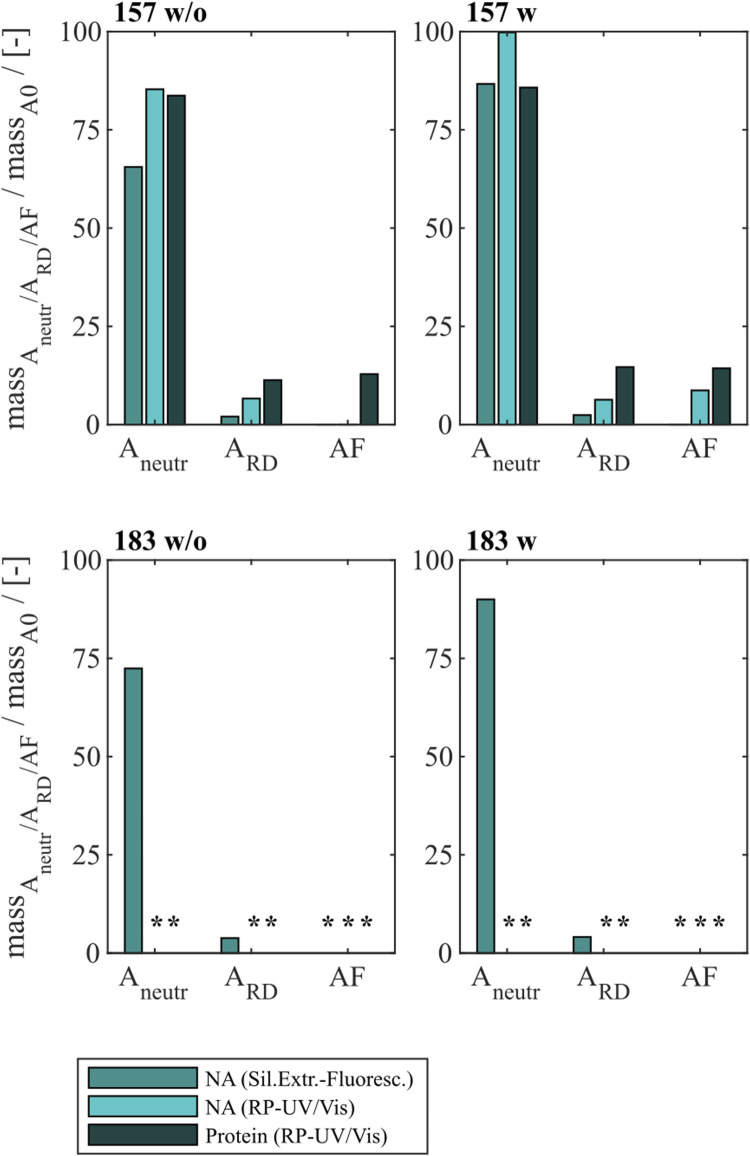
VLP protein and nucleic acid recoveries for Cp157 and Cp183 after removal of host cell-derived nucleic acids by alkaline treatment (dialysis in pH 12 buffer) with (w) and without (w/o) preliminary dialysis in 7 M Urea and NaCO_3_ solutions, and dialysis in neutralisation buffer (intermediate sample), precipitation and redissolution (intermediate sample) and size-exclusion chromatography. Nucleic acid recoveries were analysed by silica-SC based chromatography followed by dye-based fluorescence assay and RP chromatography coupled with UV/Vis analysis. VLP protein recoveries were determined by RP chromatography coupled with UV/Vis analysis. Nucleic acid and VLP protein concentrations of Cp183 for samples after dialysis in restoration buffer and redissolution (both without and with prior preliminary dialysis), were too low for RP-UV/Vis analysis, and concentrations during SEC were too low to collect and analyse fractions (*). RP: reversed phase, SC: spin column.

Finally, an alkaline treatment for Cp157 and Cp183 was performed to assess its NA_hc_ removal performance. An overview of the initial, intermediate and final process samples analyzed is shown in Figure 2. Samples after alkaline treatment and dialysis in neutralization buffer (A1), precipitation and redissolution (A2), and samples after SEC (AF) were analyzed. Moreover, procedures with and without preliminary dialysis in 7 M Urea and NaCO_3_ solutions were performed in parallel. Chromatograms of the final SEC and fraction segmentation for analysis can be found in Supplementary Material S4. For the analysis of NA_hc_ removals and VLP protein recoveries the fraction pools were analyzed for Cp157. For Cp183, a quantitative analysis of NA_hc_ removals and VLP protein recoveries was not possible, because the VLP protein concentrations were too low for fractionation sampling during SEC. NA_hc_ removals and VLP protein recoveries are displayed in Figure 6. For the procedures with and without the preliminary dialysis similar performances were obtained, respectively. For Cp157, NA_hc_ removal after alkaline treatment and dialysis in neutralization buffer (A1) was found to be 25% without and 7% with preliminary dialysis. Hereby, VLP protein recoveries of 84% and 86% were determined, respectively. The ammonium sulfate precipitation and redissolution achieved NA_hc_ removals of around 97%, averaged for redissolution and SEC fraction samples with and without preliminary dialysis (A2/AF). However, it caused high VLP protein losses resulting in VLP protein recoveries around 13%.

For Cp183, nucleic acid and VLP protein concentrations for samples after dialysis in neutralization buffer and precipitation/redissolution, both without and with prior preliminary dialysis procedures, were too low for RP-UV/Vis analysis, and concentrations during SEC were too low to collect and analyse fractions. However, the analysis by silica-SC based chromatography followed by dye-based fluorescence assay resulted in NA_hc_ removals of 18% without and 10% with preliminary dialysis after alkaline treatment and dialysis in neutralization buffer (A1). NA_hc_ removals of 96% both without and with prior preliminary dialysis in SEC fraction samples (A2) were achieved and a similar behaviour of Cp183 and Cp157 was observed. The analysis of VLP protein purity by A260/A280 ratios listed in Table 3 supports these findings by showing low VLP protein purities in the SEC fractions for both Cp157 and Cp183 with and without preliminary dialysis respectively. Further, SDS-PAGE and NAGE analyses were performed for all process samples to qualitatively track the VLP proteins and nucleic acids in the respective samples. Gel scans are depicted in Supplementary Material S4. Gel-derived results support the findings for NA_hc_ removals and VLP protein recoveries described above. Interestingly, for Cp157, NAGE analysis showed shifted VLP lanes and proteins in the gel pockets, as well as free nucleic acid lanes and nucleic acids in the gel pockets for the samples after alkaline treatment and dialysis in neutralization buffer (A1). On the contrary, in the initial sample Cp157 capsid proteins and nucleic acids were depicted at equal positions in the gel. In sum, alkaline treatment demonstrated high VLP protein losses during the process, as well as insufficient NA_hc_ removals resulting in low purities of A260/A280 ratios between 1.63 and 2.00 compared to ratios between 0.55 and 0.74 for all heparin chromatography-based processes.

**TABLE 3:** A260/A280 ratio of Cp157 and Cp183 samples after removal of host cell-derived nucleic acids bound to HBcAg VLPs alkaline treatment (without (w/o) or with (w) preliminary dialysis in 7 M Urea and NaCO_3_ solutions), intermediate samples and initial material. Ratios were determined by analysis of selected chromatography peak areas at 260 nm and 280 nm, or by microvolume UV/Vis absorbance measurements for initial and intermediate samples.

	Preliminary dialysis	A260/A280 [-]							
		Cp157				Cp183			
Alkaline treatment		initial	neutr	RD	SEC frac	initial	neutr	RD	SEC frac
	w/o	1.36	1.37	>2	1.85	1.87	1.77	>2	1.83
	w	1.36	1.43	>2	2.00	1.87	1.71	>2	1.63

## 4 Discussion

For gene delivery applications, the naturally occurring nucleic acid (NA) binding region of the hepatitis B core antigen (HBcAg) is commonly utilized for effective binding of various types of therapeutic nucleic acids (NA_ther_). During formation of the HBcAg virus-like particles (VLPs) in the *E. coli* cells host cell-derived nucleic acids (NA_hc_) associate to the NA binding region and obstruct the desired binding of NA_ther_ during loading (Birnbaum and Nassal, 1990; Newman et al., 2009; Porterfield et al., 2010). An effective removal of the undesired NA_hc_ bound to the HBcAg VLP NA binding region is essential.

### 4.1 Heparin chromatography with different HBcAg VLP constructs

In this work, a novel heparin chromatography-based process for an effective removal of NA_hc_ bound to HBcAg VLPs is presented. The VLPs were disassembled, purified by size-exclusion chromatography (SEC) and loaded onto the heparin chromatography media with disassembly buffer as running buffer. Bound material was eluted via a salt gradient. Six HBcAg VLP constructs with different lengths of the NA binding region and NA_hc_ loadings (Valentic et al., 2022) were investigated in terms of NA_hc_ removal performance by heparin chromatography.

**FIGURE 7:**
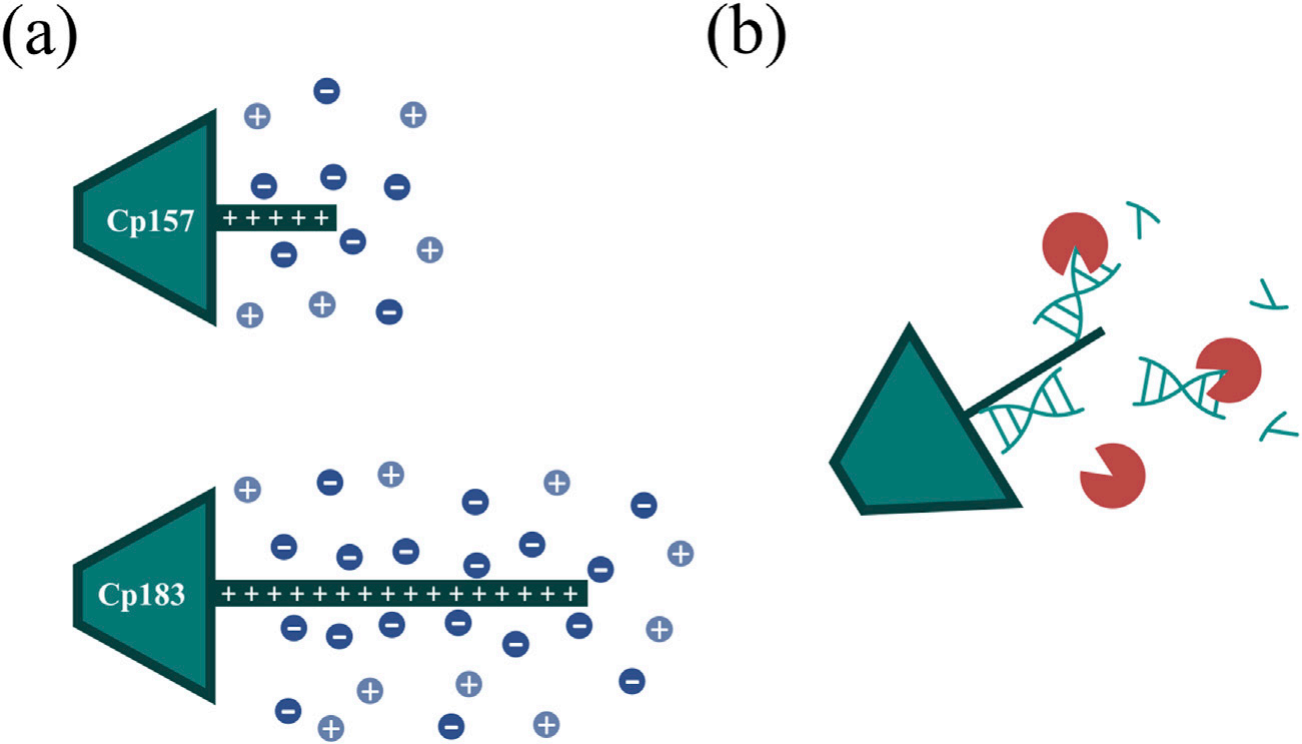
**(A)** Ionic stabilization of the NA binding region of the Cp157 and Cp183 HBcAg VLP constructs after removal of NA_hc_ by NaCl. **(B)** Degradation of NA parts bound to the NA binding region by nucleases impeded by steric hindrance. HBcAg: hepatitis B core antigen, NA: nucleic acids, NA_hc_: host cell-derived nucleic acids, VLP: virus-like particle.

Hereby, the different VLP constructs showed binding to the heparin chromatographic media dependent on the presence and length of the NA binding region and we assume the binding of the HBcAg VLP dimers to the heparin chromatographic ligands by the NA binding region, as shown in literature (Broos et al., 2007). For Cp149 and Cp154, the interactions between the affinity chromatography and the VLP proteins are too weak to bind to the column under the conditions investigated, demonstrated by SDS-PAGE analysis of the flow-through and elution fractions. Constructs with intermediate and full length of the NA binding region bind to the heparin media and are eluted with a NaCl gradient. Thereby, the NA_hc_ bound to the protamine-like NA binding region dissociate from the VLP protein and are replaced by the chromatographic ligands during loading. The replaced NA_hc_ elute in the flow-through during chromatography. Subsequent elution with the NaCl gradient weakens electrostatic interactions between the bound VLP proteins and chromatography media, leading to elution of these VLP proteins and stabilization of the VLP protein with free NA binding region by the NaCl (Figure 7A). Depending on the construct, this replacement and thus removal of NA_hc_ from the NA binding region was successful to varying degrees in terms of NA_hc_ removal and VLP protein recovery.

For the constructs Cp149 and Cp154 with no or short NA binding regions, low VLP protein recoveries were detected due to the poor binding to the heparin chromatographic media. The length of the NA binding region of Cp154 seems to be too short to effectively bind to the media. The partial binding of Cp149, lacking the NA binding region, could be explained by electrostatic attractions of positively charged amino acid residues of the dimers with the negatively charged stationary phase. This indicates a weak general binding independent from the NA binding region for all constructs. The high NA_hc_ removals determined for Cp149 may be caused by removal of free NA_hc_ in the samples. However, the already low A260/A280 ratio of the Cp149 initial material was not improved by the heparin chromatography step, confirming the non-specific binding of Cp149 to the stationary phase. Although the A260/A280 ratio for Cp154 decreased as we expected for a construct with a short NA binding region, the extent of reduction was insufficient. For the constructs Cp164, Cp167 and Cp183 we expected strong binding of the NA binding region. However, based on SDS-PAGE results and A260/A280 ratios moderate NA_hc_ removals were found. VLP protein recoveries determined by RP chromatography coupled with UV/Vis analysis revealed high VLP protein losses. The reported solubility issues of NA_hc_-free Cp183 (Porterfield et al., 2010) suggest potential post-elution aggregation or precipitation of the VLP proteins, likely due to the absence of ionic stabilization of the NA binding region (Figure 7A). However, the recovered Cp183 proteins showed high NA_hc_ removal, whereas Cp164 and Cp167 showed poor NA_hc_ removal, which needs to be further investigated in the future. For Cp157, high NA_hc_ removal accompanied by high VLP protein recovery was detected. The intermediate length of the NA binding region results in effective binding and exchange of NA_hc_ and, in addition, sufficient ionic strength stabilization of the NA binding region during and after elution. This results in high NA_hc_ removal and high VLP protein recoveries. The heparin chromatography-based NA_hc_ removal process enables binding of NA_ther_ to the NA binding region and effective loading of the HBcAg VLPs with NA_ther_ for its therapeutic application.

### 4.2 Comparison of Cp157 and Cp183

Cp157 consistently resulted in higher VLP protein recoveries than Cp183, when comparing NA_hc_ removal performances for Cp157 and Cp183 by the different investigated chromatography-based techniques, LiCl precipitation, and alkaline treatment. This substantiates the reported solubility issues of NA_hc_-free Cp183 (Porterfield et al., 2010) independent on the conducted removal process. Moreover, this points to a complex interplay between the length of the protamine-like structure and factors such as the presence of bound nucleic acids or required ions to stabilize the VLP proteins (Figure 7A), already indicated by a charge balance hypothesis for HBcAg VLP capsid stability study by Newman et al., 2009. Cp157 with an intermediate length of the NA binding region seems to be less susceptible to aggregation or precipitation after NA_hc_ removal. The determined NA_hc_ removals were on similar levels for Cp157 and Cp183. A260/A280 ratios were however consistently higher for Cp183 than for Cp157 samples for all investigated NA_hc_ removal techniques. Hence, the NA_hc_ removal appears to be more difficult with the longer NA binding region due to stronger attraction forces between the positive charges within the NA binding region and the negatively charged nucleic acids. Cp157 outperformed Cp183 in terms of NA_hc_ removal and VLP protein recovery. Thus, Cp157 is not only the superior construct for effective processing, but also likely to lead to higher NA_ther_ payloads and therefore higher therapeutic efficacy, accompanied with fewer safety issues and side effects due to NA_hc_ still present in the VLPs.

### 4.3 Comparison of heparin and sulfate chromatography

Heparin is a natural product derived from animal tissues, thus its properties can vary between batches, leading to inconsistencies in purification results and regulatory compliance issues (Liu et al., 2009; Fu et al., 2013; Baytas and Linhardt, 2020). As an alternative, sulfate chromatography (Begic et al., 2021; Steppert et al., 2022) was investigated next to heparin chromatography for an effective NA_hc_ removal for Cp157 and Cp183. Moreover, effects of a prior nuclease treatment on the removal performance of both chromatography-based processes were assessed. Both heparin and sulfate chromatography resulted consistently in high NA_hc_ removals. Sulfate chromatography appears to be a suitable heparin alternative for NA_hc_ removal. The observed drawbacks of sulfate in terms of VLP protein recovery compared to heparin chromatography are only comparable to a limited extent due to different stationary phase volumes used in the investigations presented. Material in high salt washes during the chromatography uncovered VLP protein loss due to strong binding to the stationary matrix. For a sufficiently meaningful comparison of VLP protein recovery for heparin and sulphate chromatography, experiments would have to be performed using a comparable chromatography column volume.

To evaluate effects of nuclease treatment on removal performance parameters such as NA_hc_ removal and VLP protein recovery we aimed to apply quantitative analyses. In our study, nuclease treatment prior to the performed chromatography-based processes improved NA_hc_ removal marginally. Our hypothesis here is, that only–to a large extend - NA regions that are not covered by the NA binding region are degraded. Degradation of NA parts bound to the NA binding region may not be effective due to steric hindrance of the enzymatic reaction (Figure 7B). The degradation of nucleic acids may also have little effect on the proposed replacement of nucleic acids by ligands of the applied chromatography media. Additionally, we observed challenges in the removal of the nucleases in the subsequent downstream process. Nuclease removal is, however, essential to prevent degradation of the therapeutic nucleic acids (NA_ther_) after loading. An ultrafiltration step could be successful to separate the nucleases prior to loading (Hillebrandt et al., 2020). However, this additional step would require development and would reduce the VLP protein yield (Hillebrandt and Hubbuch, 2023).

### 4.4 LiCl precipitation and alkaline treatment

LiCl precipitation and alkaline treatment techniques were reported to remove NA_hc_ from Cp183 (Porterfield et al., 2010; Petrovskis et al., 2021). This was qualitatively evaluated by transmission electron microscopy and native agarose gel electrophoresis (NAGE) analysis. In order to quantitatively compare the heparin chromatography and process variants presented with LiCl precipitation and alkaline treatment with respect to their NA_hc_ removal performance, LiCl precipitation and alkaline treatment were performed with Cp157 and Cp183. Nucleic acid quantification by RP-UV/Vis of intermediate LiCl precipitation samples after centrifugation was not possible due to the presence of guanidine HCl in the samples, overlapping with the nucleic acid peaks in the flow-through of the RP-HPLC method. Therefore, it was decided not to average the NA_hc_ removals obtained by the two available analytical methods across the study. The NA_hc_ removal by LiCl precipitation from Cp183 has been evaluated by A260/A280 ratio determination at the SEC (Porterfield et al., 2010), resulting in final ratios of 0.6. However, VLP protein recoveries were not reported, which would be required for a final validation of NA_hc_ removal suitable for effective processing. In contrast, our study found a poor A260/A280 ratio of 1.74 for Cp183 in SEC and very low VLP protein recoveries. Cp157 resulted in slightly better NA_hc_ removals and VLP protein recoveries, with a final A260/A280 ratio of 0.95. However, these findings exhibit notably inferior performance of LiCl precipitation when contrasted with all chromatography-based NA_hc_ removal processes presented. Intermediate samples during the LiCl precipitation process were analyzed to identify process steps causing this protein loss. However, this analysis was inconclusive. The RP-UV/Vis determined still high VLP protein recoveries after the centrifugation, whereas SDS-PAGE analysis indicates significant VLP protein losses during the LiCl precipitation and centrifugation step. There may be unknown effects of the components in the disassembly and precipitation buffer used onto the RP-UV/Vis method, that not only interfere with nucleic acid quantification, but also affect protein quantification by this method. We suspect an insufficient separation of the VLP proteins and bound NA_hc_, resulting in co-precipitation of the VLP proteins and low VLP protein recoveries.

So far, the NA_hc_ removal by alkaline treatment from Cp183 has been shown qualitatively by NAGE (Strods et al., 2015). However, quantitative analysis of the VLP protein recovered and NA_hc_ removed required for effective processing was not reported. Evaluation of NA_hc_ removal performance for Cp157 and Cp183 in our study revealed high VLP protein losses during the alkaline treatment process. Due to the already low initial concentration of the Cp183 protein, quantification of nucleic acid and VLP protein by RP-UV/Vis was not possible, thus precluding a comprehensive quantitative evaluation of the various steps involved. However, SDS-PAGE and NAGE analyses of Cp183 are in qualitative agreement with the results for Cp157. Cp157 resulted in better performance characteristics after the alkaline treatment procedures than Cp183, as with the other NA_hc_ removing approaches studied. The alkaline treatment resulted in minor VLP protein losses, both with and without the preliminary dialysis in 7 M Urea and NaCO_3_ solutions suggested by Strods et al., 2015. The long dialysis times in high pH buffers impaired VLP protein solubility less than we expected. However, only moderate NA_hc_ removals were achieved after alkaline treatment and dialysis in neutralization buffer. The dialysis in pH 12 buffer may decrease the electrostatic attractions between the NA binding region and bound NA_hc_ by deprotonating guanidinium groups of the arginines within the NA binding region (Hunter and Borsook, 1924; Schmidt et al., 1930). The detached nucleic acids may be separated by dialysis in pH 12 buffer or the subsequent processing steps. Potential reasons for the inefficacious removal of NA_hc_ by dialysis in alkaline buffer include the possibility that pH 12 might not alkalize sufficiently to induce deprotonation of the guanidinium group of arginine (Fitch et al., 2015). Alternatively, it is plausible that the unbound NA_hc_ molecules remained entrapped within the VLP capsids and consequently could not be effectively separated via dialysis. However, in contrast to the latter hypothesis, NAGE analysis showed free nucleic acids in the sample, indicating that the nucleic acids are no longer entrapped in the VLPs and need to be separated in further purification steps. Omission of the preliminary dialysis in 7 M Urea and NaCO_3_ solutions and direct alkaline treatment by dialysis in pH 12 buffer resulted in more successful removal performances. Given the ambiguous rationale behind the initial dialysis step in the alkaline treatment described by Strods et al., 2015, and the absence of significant disparities in VLP protein and nucleic acid attributes using SDS-PAGE and NAGE between samples subjected to preliminary dialysis and those not, discerning potential explanations for these outcomes is challenging. Further separation of free nucleic acids was performed by precipitation, redissolution and SEC. The process of precipitation and subsequent redissolution led to notable losses of VLP protein and requires refinement for enhanced efficiency in processing. Nevertheless, examination of A260/A280 ratios of target fractions in SEC indicated that the alkaline treatment was insufficient in effectively removing NA_hc_.

In summary, the chromatography-based processes presented demonstrated effective removal of NA_hc_ bound to HBcAg VLPs, required for subsequent binding of NA_ther_, likely resulting in high NA_ther_ payloads and thus therapeutic efficacy. Through a comprehensive comparison of various process variants employing heparin and sulfate chromatography, both with and without prior nuclease treatments, along with novel quantitative assessments of NA_hc_ removals and VLP protein recoveries reported earlier (Valentic et al., 2024), it has been shown that the performance of LiCl precipitation and alkaline treatment is insufficient when compared to our proposed chromatography-based approaches. Among these approaches, the highest levels of NA_hc_ removal and VLP protein recovery were achieved for Cp157 using heparin chromatography without prior nuclease treatment.

## 5 Conclusion

In conclusion, this study presents a novel heparin chromatography-based process for the effective removal of NA_hc_ bound to HBcAg VLPs. This is required both to prevent potential side effects due to the presence of NA_hc_ and to subsequently load the VLPs with NA_ther_. The process was evaluated using six different HBcAg VLP constructs with varying lengths of the NA binding region and different NA_hc_ loadings. Process performance was assessed through NA_hc_ removal and VLP protein recovery analyses using RP chromatography coupled with UV/Vis spectroscopy, and silica-SC based chromatography combined with a dye-based fluorescence assay. Additionally, alternative process variants such as nuclease treatments and sulfate chromatography, as well as LiCl precipitation and alkaline treatment procedures were also explored for comparison with the newly developed chromatography-based method. The investigation of the heparin chromatography process using different HBcAg VLP constructs revealed a nuanced relationship between NA binding region length and NA_hc_ removal efficiency, with Cp157 exhibiting optimal performances. While slight differences were observed among process variants for the selected constructs Cp157 and Cp183, the heparin chromatography-based process consistently outperformed LiCl precipitation and alkaline treatment methods in terms of NA_hc_ removal and VLP protein recovery, highlighting its superiority over these techniques. Finally, the presented method might act as a blueprint for other vector systems where NA_hc_ removal is an issue.

## Data Availability

The raw data supporting the conclusions of this article will be made available by the authors, without undue reservation.

## Abbreviations

HBcAg, hepatitis B core antigen; HPV, human papillomavirus; NA, nucleic acid; NAhc, host cell-derived nucleic acids; NAther, therapeutic nucleic acids; NAGE, native agarose gel electrophoresis; NT, nuclease treatment; RP, reversed phase; SEC, size-exclusion chromatography; SC, spin column; VLPs, virus-like particles.
