# Supplementary material for: Effective removal of host cell-derived nucleic acids bound to hepatitis B core antigen virus-like particles by heparin chromatography
Source: Front Bioeng Biotechnol. 2024 Oct 3;12:1475918. doi: 10.3389/fbioe.2024.1475918 (PMC11487522; doi:10.3389/fbioe.2024.1475918)
Supplement: Supplementary file 1 [file DataSheet1.PDF]

S1 SDS-PAGE analysis of heparin chromatography for different HBcAg VLP constructs

Table S1.1: Overview samples investigated by SDS-PAGE for the different HBcAg VLP constructs during heparin chromatography. M: marker, I: initial sample, FT: flow-through, E: elution

Lane	**1**	**2**	**3**	**4**	**5**	**6**	**7**	**8**	**9**	**10**	**11**	**12**
Cp149	M	I	FT 1	FT 2	FT 3	E 1	E 2	E 3	E 4	-	-	-
Cp154	M	I	FT 1	FT 2	FT 3	E 1	E 2	E 3	E 4	-	-	-
Cp157	M	I	E 1	E 2	E 3	E 4	E 5	FT 1	FT 2	FT 3	FT 4	FT 5
Cp164	M	I	FT 1	FT 2	FT 3	FT 4	E 1	E 2	E 3	E 4	-	-
Cp167	M	I	FT 1	FT 2	FT 3	FT 4	E 1	-	E 2	-	E 3	E 4
Cp183	M	FT 1	FT 2	FT 3	FT 4	E 1	E 2	E 3	E 4	E 5	I	-

Table S1.2: Gel scans of SDS-PAGE analysis of flow-through and elution fractions of heparin chromatography of HBcAg VLP constructs Cp149, Cp154, Cp157, Cp164, Cp167, and Cp183.

Cp149

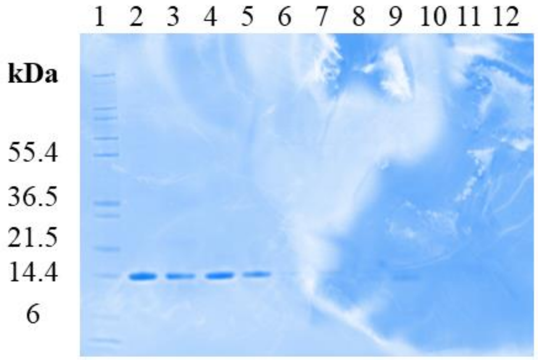

Cp154

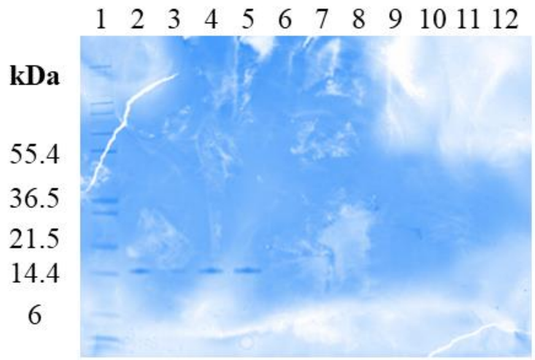

Cp157

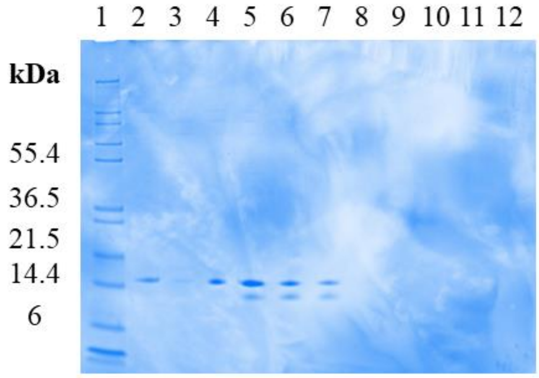

Cp164

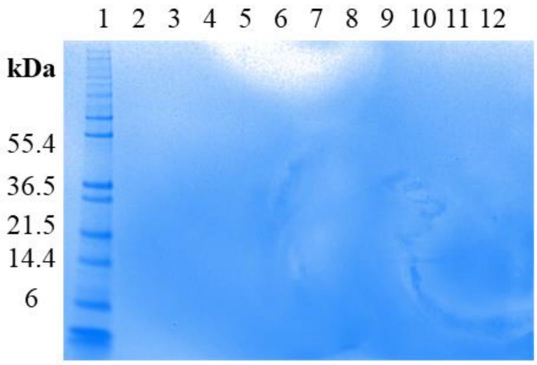

Cp167	Cp183
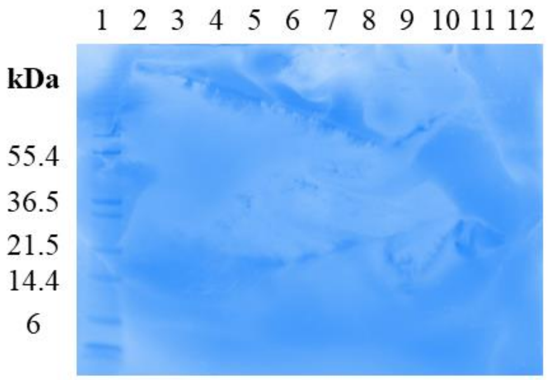	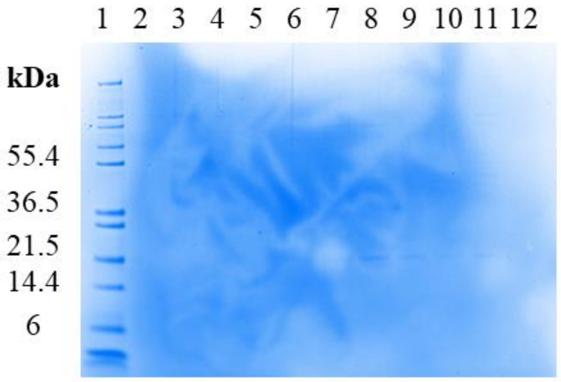

S2 SDS-PAGE analysis of heparin and sulfate chromatography with and without prior nuclease treatment

Table S2.1: Overview of samples investigated by SDS-PAGE for HBcAg VLP constructs Cp157 and Cp183 and heparin and sulfate chromatography with and without prior nuclease treatment. M: marker, I: initial sample, FT: flow-through, E: elution, +: with nuclease treatment

Lane	**1**	**2**	**3**	**4**	**5**	**6**	**7**	**8**	**9**	**10**	**11**	**12**
Cp157 Heparin with nuclease	M	I	E 1	E 2	E 3	E 4	E5	FT 1	FT 2	FT 3	FT 4	FT 5
Cp157 Sulfate without nuclease	M	I	FT 1	FT 2	FT 3	FT 4	E 1	E 2	E 3	E 4	W	-
Cp157 Sulfate with nuclease	M	I	FT 1	FT 2	FT 3	E 1	E 2	E 3	E 4	-	W	-
Cp183 Heparin without nuclease	M	FT 1	FT 2	FT 3	FT 4	E 1	E 2	E 3	E 4	E 5	I	-
Cp183 Heparin with nuclease	M	FT 1	FT 2	FT 3	FT 4	E 1	E 2	E 3	E 4	E 5	I	-
Cp183 Sulfate with (+) / without nuclease	M	FT 1	FT 2	FT 3	E 1	E 2	E 3	FT 1 +	FT 2 +	FT 3 +	E 1 +	I

Table S2.2: Gel scans of SDS-PAGE analysis of flow-through and elution fractions of heparin and sulfate chromatography with and without prior nuclease treatment and HBcAg VLP constructs Cp157 and Cp183.

**Cp157; heparin with nuclease**	**Cp157; sulfate without nuclease**
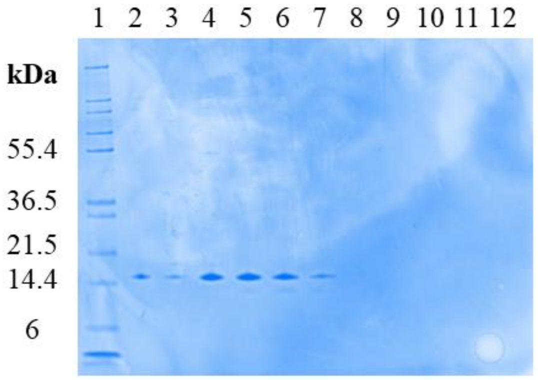	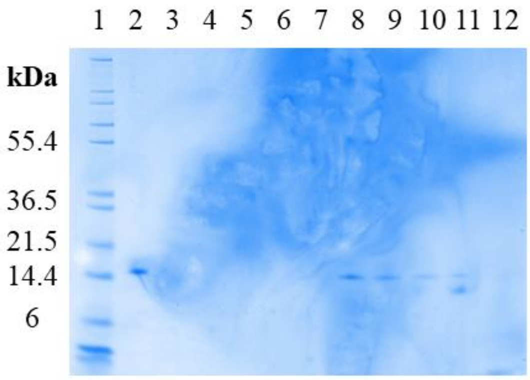
**Cp157; sulfate with nuclease**	**Cp183; Heparin without nuclease**
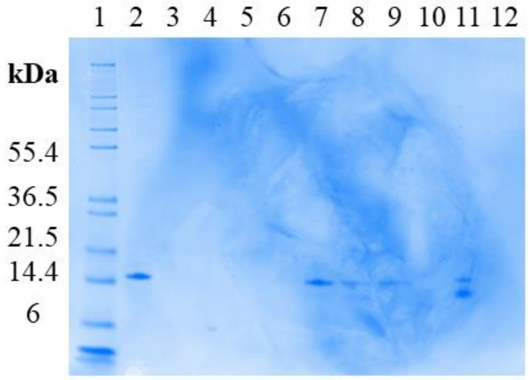	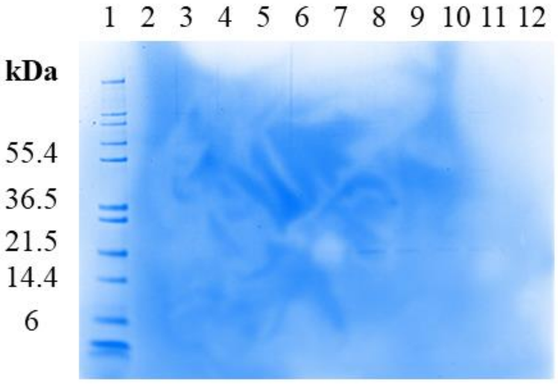
**Cp183; heparin with nuclease**	**Cp183; sulfate without/with (+) nuclease**
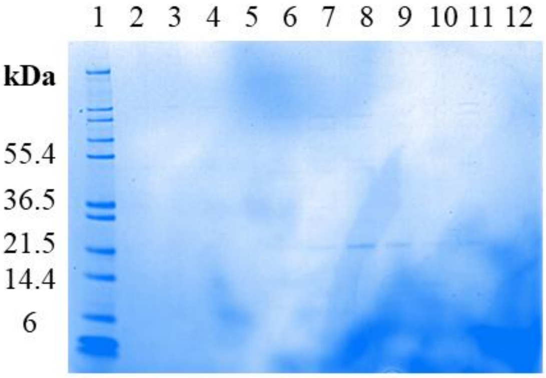	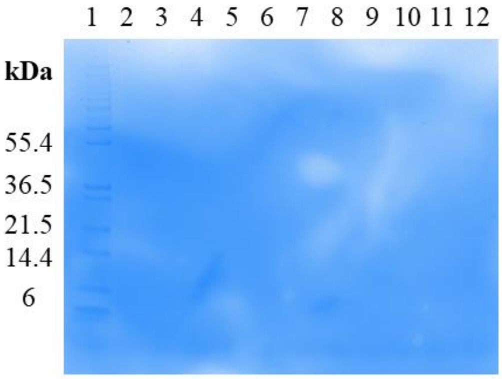

S3 SEC chromatograms and gel electrophoresis analysis of LiCl precipitation

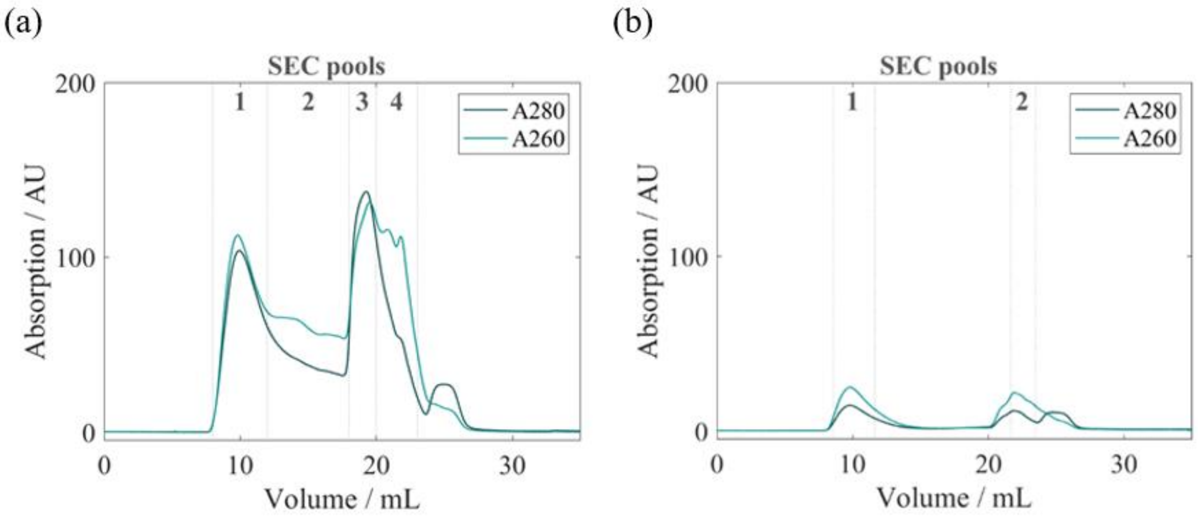

Figure S3.1: Chromatograms of the final SEC and fraction segmentation for analysis of host cell-derived nucleic acid removal by LiCl precipitation for (a) Cp157 and (b) Cp183.

Table S3.1: Overview of samples investigated by SDS-PAGE for HBcAg VLP constructs Cp157 and Cp183 during LiCl precipitation. M: marker, I: initial sample, C: after centrifugation, SEC: size-exclusion chromatography pools

Lane	**1**	**2**	**3**	**4**	**5**	**6**	**7**	**8**	**9**	**10**	**11**	**12**
Cp157	M	I	I	C	SEC 1	SEC 2	SEC 3	SEC 4	-	-	-	-
Cp183	M	I	I	C	SEC 1	SEC 2	-	-	-	-	-	-

Table S3.2: Gel scans of SDS-PAGE analysis of HBcAg VLP constructs Cp157 and Cp183 during LiCl precipitation.

**Cp157**	**Cp183**
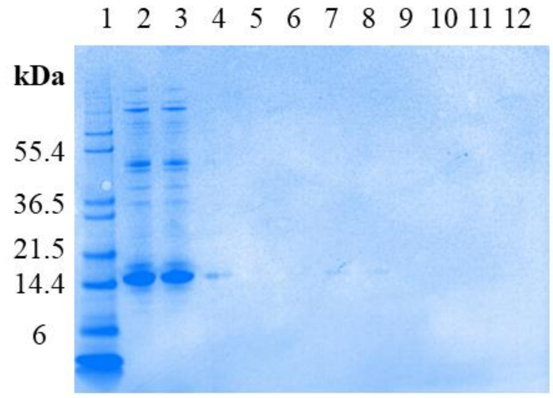	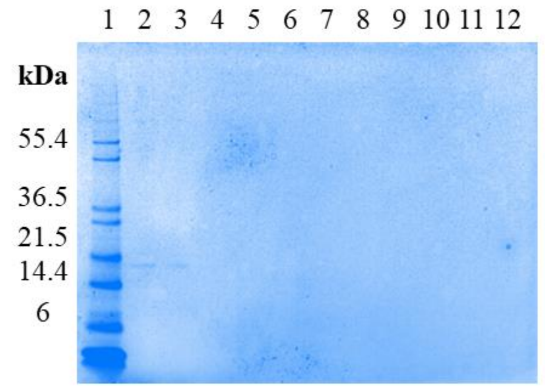

Table S3.3: Overview of samples investigated by NAGE for HBcAg VLP constructs Cp157 and Cp183 during LiCl precipitation. M: marker, I: initial sample, C: after centrifugation, SEC: size-exclusion chromatography pools

1	2	3	4	5	6	7	8	9	10	11	12	13
Cp157 I	Cp157 I	Cp157 C	Cp157 SEC 1	Cp157 SEC 2	Cp157 SEC 3	Cp157 SEC 4	-	Cp183 I	Cp183 I	Cp183 C	Cp183 SEC 1	Cp183 SEC 2

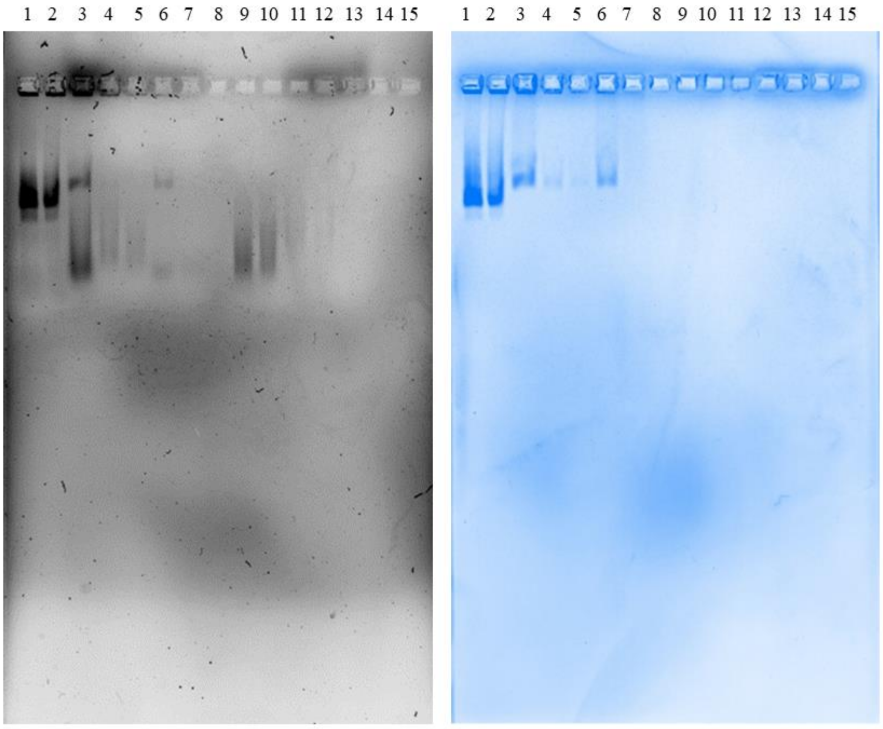

Figure S3.2: Gel scans of NAGE analysis of HBcAg VLP constructs Cp157 and Cp183 during LiCl precipitation by midori green (left) and Coomassie (right).

S4 SEC and gel electrophoresis analysis of alkaline treatment

Table S4.1: Overview of samples after dialysis in neutralization buffer investigated by SDS-PAGE for HBcAg VLP constructs Cp157 and Cp183 during alkaline treatment. M: marker, I: initial sample, w: with preliminary dialysis, w/o without preliminary dialysis

1	2	3	4	5	6	7	8	9
M	Cp157 I	Cp157 w treatment	Cp157 w/o treatment	-	Cp183 I	Cp183 w treatment	Cp183 w/o treatment	-

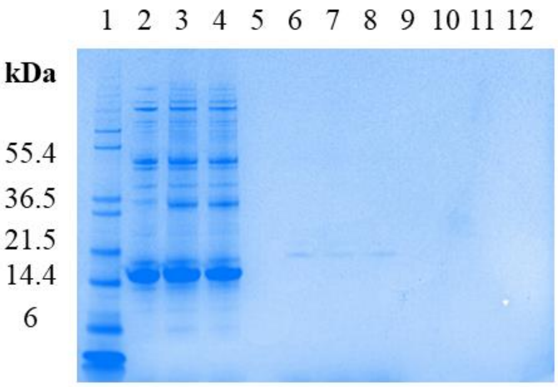

Figure S4.1: Gel scan of SDS-PAGE analysis of HBcAg VLP constructs Cp157 and Cp183 after dialysis in neutralization buffer during alkaline treatment.

Table S4.2: Overview of samples after dialysis in neutralization buffer investigated by NAGE for HBcAg VLP constructs Cp157 and Cp183 during alkaline treatment. M: marker, I: initial sample, w: with preliminary dialysis, w/o without preliminary dialysis

1	2	3	4	5	6	7	8
Cp157 I	Cp157 w treatment	Cp157 w/o treatment	-	Cp183 I	Cp183 w treatment	Cp183 w/o treatment	-

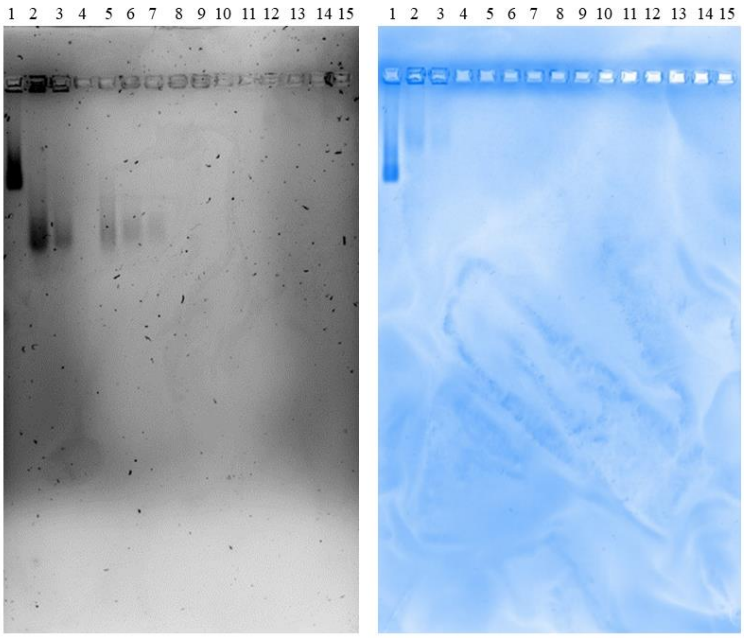

Figure S4.2: Gel scans of NAGE analysis of Cp157 and Cp183 samples after dialysis in neutralization buffer during alkaline treatment by midori green (left) and Coomassie (right).

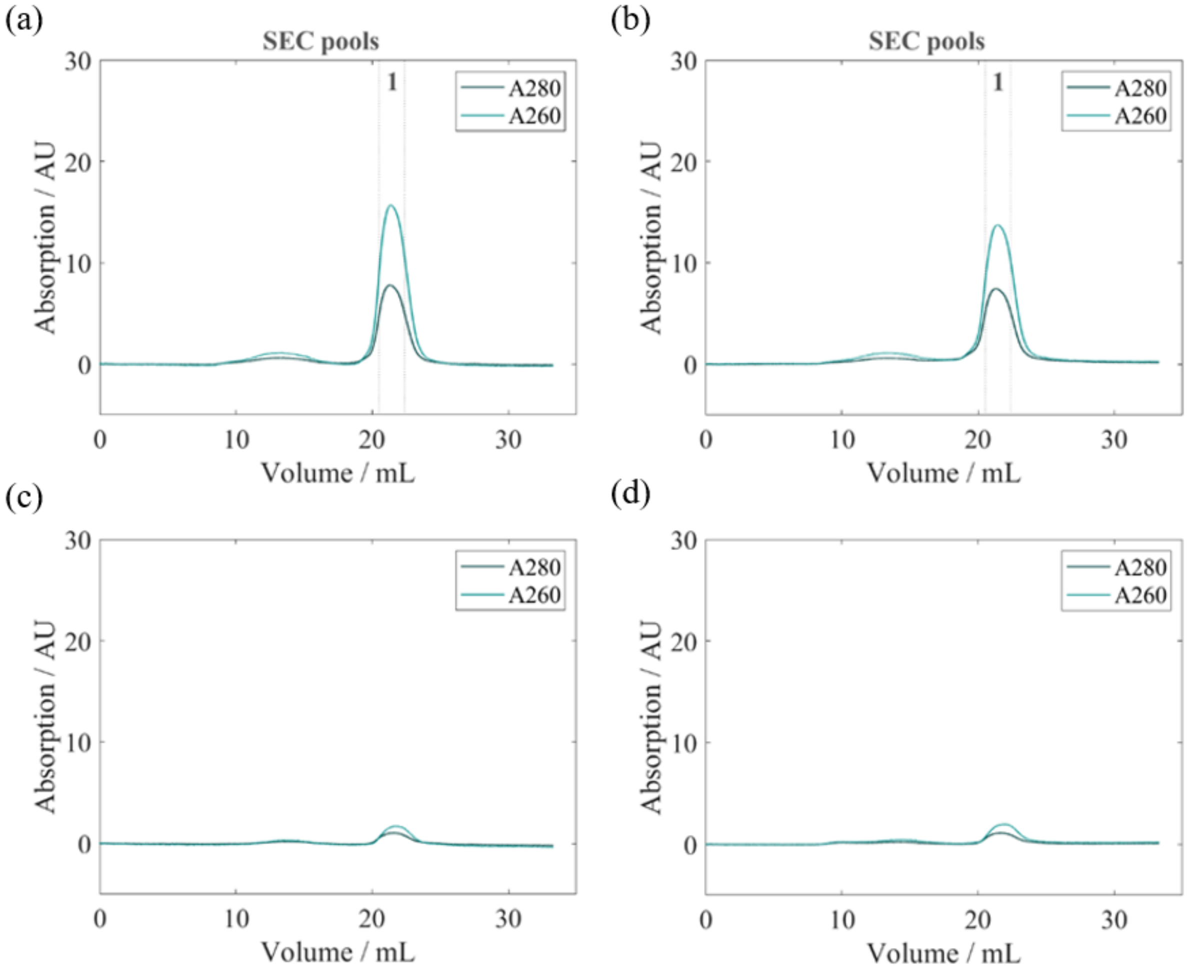

Figure S4.3: Chromatograms of the final SEC and fraction segmentation for analysis of host cell-derived nucleic acid removal by alkaline treatment for Cp157 with (a) and without (b), and Cp183 with (c) and without (d) preliminary dialysis.

Table S4.3: Overview of samples after redissolution and SEC fractions investigated by SDS-PAGE and NAGE for HBcAg VLP constructs Cp157 and Cp183 during alkaline treatment. M: marker, I: initial sample, w: with, w/o without, PT: preliminary treatment, RD: redissolution

1	2	3	4	5	6	7	8	9	10
Cp157 I	Cp157 w PT RD	Cp157 w/o PT RD	Cp157 w PT SEC 1	Cp157 w/o PT SEC 1	-	Cp183 I	Cp183 w PT RD	Cp183 w/o PT RD	-

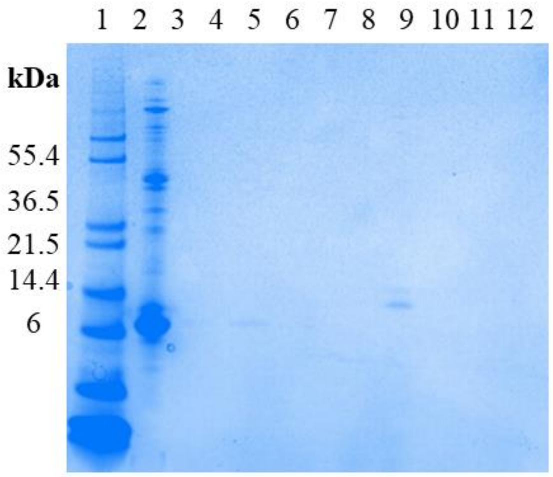

Figure S4.4: Gel scan of SDS-PAGE analysis of Cp157 and Cp183 samples after redissolution and SEC fractions during alkaline treatment.

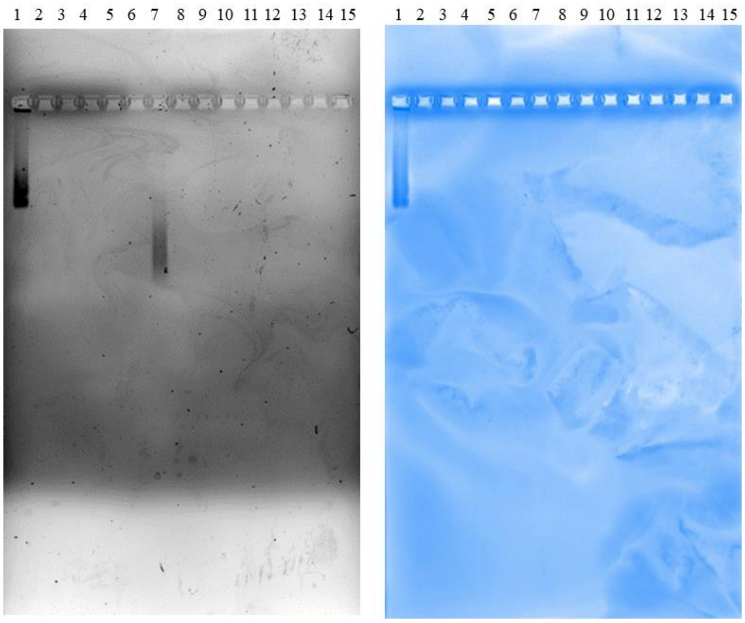

Figure S4.5: Gel scans of NAGE analysis Cp157 and Cp183 samples after redissolution and SEC fractions during alkaline treatment by midori green (left) and Coomassie (right).
